# Adherence to artemether-lumefantrine drug combination: a rural community experience six years after change of malaria treatment policy in Tanzania

**DOI:** 10.1186/1475-2875-13-S1-P62

**Published:** 2014-09-22

**Authors:** Omary Minzi, Sylivester Maige, Philip Sasi, Billy Ngasala

**Affiliations:** 1 Muhimbili University of Health and Allied Sciences, Dar es Salaam, Tanzania

## Background

Adherence to multidosing is challenging worldwide. This study assessed the extent of adherence to multidosing artemether-lumefantrine (ALu) in a rural community in Tanzania, six years after switching from single dose policy of sulphadoxine-pyrimethamine.

## Materials and methods

This study was a prospective observational, open label, non-randomized study involving 151 patients with un-complicated malaria recruited at Fukayosi dispensary in Bagamoyo district in Tanzania. Patients treated with ALu were visited at home on day 3 for interview on drug intake, capillary blood sample collection for microscopy and ALu tablets count. Venous blood samples (2 ml) for determination of blood lumefantrine concentrations and blood slides for microscopy were collected on day 7. Kappa’s coefficient was used to assess agreement between pill count and self-report. Adherence was categorized depending on the tablets remaining and what the patient reported. Only those with empty blister pack available but no tablet remaining and reported taking all six doses of ALu at a correct dose and correct time were regarded as definite adherent. The rest were either probable adherent or probable non-adherent.

## Results

Only 14.9% of the patients were definite adherent, the rest took the drug at incorrect time or did not finish the tablets. Out of 90 patients with analyzed plasma samples for lumefantrine blood concentrations, 13/90 (14.4.0%) had lumefantrine concentrations <175 ng/ml. There was no difference in mean lumefantrine concentration in the patients who stated to have taken all doses as required (561.61 ng/ml 95% CI = 419.81-703.41) compared to those who stated to have not adhered well to drug intake (490.95 ng/ml, 95% CI = 404.18-577.7074 (*P* = 0.643). None of the patients had detectable parasites by microscopy on day 3 and day 7 regardless of adherence status and the level of day 7 blood lumefantrine. There was strong agreement between the self-reported responses on drug intake and pill-counts (kappa coefficient = 0.955). Age, sex, education and place where first dose was taken were associated with adherence.

## Conclusion

The overall adherence 6 years after the change of malaria treatment policy was low. It is, therefore, important to continuously monitor the level of adherence to treatment in order to get the current situation and institute corrective measures on time.

